# Vincent’s Angina—Sequellae

**Published:** 1916-11

**Authors:** 


**Vincent's Angina—Sequellae.** T. H. Glenn and W. R. Dodge, Fort Dodge, Ia., St. Paul Med. Jour., Sept. Among 8 cases seen in a year, one syphilitic developed a membrane over the whole buccal cavity, gastro-intestinal symptoms and death. The other developed multiple paralyses as after diphtheria which was excluded bacteriologically as was syphilis by a negative Wasserman—Recovery.

